# Comparison of the proliferation, cytotoxic activity and cytokine secretion function of cascade primed immune cells and cytokine-induced killer cells *in vitro*

**DOI:** 10.3892/mmr.2015.3765

**Published:** 2015-05-08

**Authors:** GUI-XIN LI, SHU-SHU ZHAO, XU-GUANG ZHANG, WEN-HAO WANG, JIN LIU, KE-WEI XUE, XIAO-YAN LI, YING-XUE GUO, LI-HUA WANG

**Affiliations:** 1 Department of Oncology, Affiliated Hospital of Weifang Medical University, Weifang, Shandong 261031, P.R. China; 2 Department of Clinical Laboratory, Affiliated Hospital of Weifang Medical University, Weifang, Shandong 261031, P.R. China

**Keywords:** cascade primed immune cells, cytokine-induced killer cells, proliferation, cytotoxic activity, cytokine

## Abstract

The present study aimed to compare the antitumor effects of cascade primed immune (CAPRI) cells and cytokine-induced killer (CIK) cells *in vitro*, through investigating cell morphology, proliferation, cytotoxic activity to tumor cells and the ability of these cells to secrete cytokines. Peripheral blood samples (50 ml) were obtained from three healthy volunteers and peripheral blood mononuclear cells (PBMCs) were obtained from each via Ficoll-Conray density gradient centrifugation. Each suspension of PBMCs (1×10^6^/ml) was divided into two parts; CAPRI cells were obtained from one part through a series of induction, amplification and cytokine cultures, while CIK cells were obtained from the other part through induction with different cytokines. During the culture process, the proliferation and morphological changes were observed for the two cell types using Trypan blue staining. At day 14, the cytotoxic activity of the two cell types was examined through determining lactate dehydrogenase release in the presence of K562 leukemia cells and MCF-7 breast cancer cells. In addition, secretory levels of interferon (IFN)-γ and interleukin (IL)-2 were detected using enzyme-linked immunospot (ELISPOT) technology. The results revealed that at day 5 and 14 of culture, there were significantly fewer CAPRI cells compared with CIK cells (P<0.001), although the survival rate of each cell type was >95%. The cytotoxic activity of CAPRI cells towards the K562 cell line was effector-target ratio-dependent (40:1 and 20:1) with values of 55.1±3.25 and 35.0±2.65%, respectively, which were significantly reduced compared with the corresponding data in CIK cells, 60.0±3.03 and 39.7±3.42% (P=0.004 and 0.005, respectively). Furthermore, the cytotoxic activity of CAPRI cells towards MCF-7 cells were 71.5±3.06, 56.0±3.76 and 40.2±2.90% at effector-target ratios 40:1, 20:1 and 10:1, respectively. These data were significantly higher than the corresponding values in CIK cells, 65.4±3.86, 49.5±3.91 and 36.1±3.73% (P=0.002, 0.003 and 0.02, respectively). As determined using ELISPOT technology at different cell concentrations (1×10^6^/ml and 5×10^5^/ml), IFN-γ secretion levels, determined by the number of spot-forming cells, of CAPRI cells were 126.2±10.31 and 48.8±10.99, respectively, which were significantly reduced compared with those of CIK cells, 409.3±7.76 and 159.3±15.45, respectively (P<0.001). IL-2 secretion levels in CAPRI cells were 325.1±16.24 and 113.8±11.29 at 1×10^6^/ml and 5×10^5^/ml, respectively, which were significantly increased compared with CIK cells, 212.0±16.58 and 70.7±10.57, respectively (P<0.001). In conclusion, the present study demonstrated that CAPRI cells had a reduced proliferation rate compared with CIK cells as well as a less potent cytotoxic effect on K562 cells; however, the two cell types had potent cytotoxic activity towards solid tumor MCF-7 cells. In addition, CAPRI cells secreted lower levels of IFN-γ and increased levels of IL-2 compared with CIK cells. These results indicated that antitumor activities of CAPRI and CIK cells proceeded via different mechanisms.

## Introduction

Cancer is a prominent public health problem worldwide, which has increasing incidence and mortality rates (1). Progress has been made in improving cancer therapy, with surgical resection, chemotherapy and radiotherapy being the three major conventional modes of cancer treatment (2). However, effective treatment remains to be achieved for numerous types of tumors (2). Biological treatment is a novel model in comprehensive cancer treatment, which has received extensive attention (3,4). Adoptive cellular immunotherapy (ACI) is an important form of biological tumor therapy, which involves the infusion of autologous or allogeneic immune cells in order to enhance immune function in patients and in turn achieve antitumor effects (5).

Cascade primed immune (CAPRI) cells and cytokine-induced killer (CIK) cells have been used as novel adoptive immunotherapy cells and are known to have different strengths and biological characteristics (6). These cells have been widely used in previous clinical studies; however, there have been no systematic comparative evaluations of the two treatments (7,8). Therefore, the present study aimed to compare the antitumor effects of CAPRI and CIK cells *in vitro*, through investigating cell morphology, proliferation, cytotoxic activity to tumor cells and the ability of these cells to secrete cytokines. These methods of comparison may be extended for the future detection of a variety of cell lines and cytokines in order to better guide clinical treatment.

## Materials and methods

### Materials and reagents

K562 human leukemia cells and MCF-7 human breast cancer cells were purchased from the cell library of Cancer Institute of Chinese Medical Sciences Academy (Beijing, China). K562 and MCF-7 cells were cultured in RPMI 1640 medium (Beijing Suolaibao Science and Technology Co., Ltd., Beijing, China) with 10% fetal bovine serum (FBS; HyClone Laboratories, Inc., Logan, UT, USA) at 37°C in 5% CO_2_. Low molecular weight heparin injections were purchased from Qilu Pharmaceutical Co., Ltd (Jinan, China). The substrate mix (BCIP/NBT Color Development Substrate) was purchased from Promega Corp. (Madison, WI, USA). 96-well plates and cell culture flasks were obtained from Beijing Aibo Biological Engineering Co., Ltd. Recombinant human (rHu) interferon (INF)-γ and rHu interleukin (IL)-2 were purchased from Peprotech Co. (Rocky Hill, NJ, USA). Mouse monoclonal anti-human CD3 antibodies were provided by Beijing Tonglihaiyuan Biotechnology Co., Ltd. (Beijing, China). CAPRI cell culture patented reagents were obtained from the Institute of Immunology, University of Munich (Munich, Germany). Lymphocyte stratified fluid was purchased from Tianjin Haoyang Biological Products Co., Ltd (Tianjin, China). IFN-γ and IL-2 ELISPOT kits were purchased from Shenzhen Dakewei Biotechnology Co., Ltd. (Guangdong, China).

### Instruments

Clean benches, portable autoclaves and the constant temperature water bath were purchased from Shanghai Boxun Industry and Commerce Co., Ltd. (Shanghai, China). The CO_2_ incubator was obtained from Sanyo Electric International Trading Co., Ltd. (Shanghai, China) and the micropipettes were purchased from Thermo Fisher Scientific Co., Ltd. (Shanghai, China). The low-speed centrifuge (B40) was obtained from Hebei Anxin Baiyang Centrifugal Machinery Factory (Hebei, China) and enzyme-linked immunospot (ELISPOT; S5) analyzers were provided by Cellular Technology, Ltd (Shaker Heights, OH, USA). The inverted microscope (BDS200) was obtained from Chongqing Auto Optical Instruments Co., Ltd. (Chongqing, China) and the ELISA analyzer (RT2100c) was obtained from Rayto Life and Analytical Science Co. (Shenzhen, China).

### Culture of CAPRI cells and CIK cells

The current study was approved by the Ethics Committee of the Affiliated Hospital of Weifang Medical University (Weifang, China) and written informed consent was obtained from the patient/the patient’s families. A total of 50 ml blood was extracted from three healthy volunteers and heparin (1 ml; 6250 U/ml) was added to prevent coagulation. The three specimens were then stratified with lymphocyte fluid. Ficoll-Conray density gradient centrifugation (50–60 ml peripheral blood, density 1.077±0.001 g/ml) was performed to obtain three separate suspensions of peripheral blood mononuclear cells (PBMCs). Following washing with 0.9% NaCl, RPMI 1640 medium was used to adjust the PBMC suspension to 1×10^6^cells/ml. Samples were labeled (a, b and c) and the PBMC suspensions from each source were divided into two parts as follows: a1, b1 and c1 for CAPRI cell induction; and a2, b2 and c2 for CIK cell induction.

### CAPRI cells induction

A total of 12 ml coating solution (Shanghai Weike Co., Ltd., Shanghai, China) was added to three flasks, which were then incubated at 4°C for 24 h. The coating solution was removed and 20 ml saline (Shandong Qidu Pharmaceutical Co., Ltd., Shandong, China) was added to each flask, which were then mixed uniformly and left to stand for 2 min prior to saline removal. A total of 18 ml complete medium (RPMI 1640 and 10% FBS) was added to each flask. PBMC suspensions (12 ml; 1×10^6^ cells/ml) of a1, b1 and c1 were added to the flasks and incubated at 37°C in a 5% CO_2_ incubator for 3 h. CAPRI patent reagent A (0.4 ml) was added to each culture flask for 3 h at 25°C and the stimulated culture substances were subsequently obtained.

Lymphocyte stratified fluid (12 ml) was added to each stimulated culture medium and then cultured at 37°C in a 5% CO_2_ incubator for 16 h. The culture substances were collected in 50 ml centrifuge tubes and centrifuged at 750 × g for 8 min. The supernatants were discarded and RPMI 1640 was used to resuspend cells. Cells were then counted using a cell counting plate under the inverted microscope and were subjected to further centrifugation (800 × g for 12 min).

Complete medium was used to resuspend the cells and CAPRI patent reagent B (0.4 ml) was added to each cell suspension, which were then dispensed into flasks and cultured at 37°C in a 5% CO_2_ incubator for 72 h. CAPRI cells were then collected and used for subsequent experiments. One part were further cultured (3×10^6^ cells/ml; −80°C) to study the cells proliferative capacity, the other groups (3×10^6^ cells/ml) were frozen (−80°C) till 14 days and the cytotoxic activity and cytokine secretion levels of them were used to perform a comparative study with CIK cells.

### CIK cells induction

The three PBMC suspensions (a2, b2 and c2; 12 ml) were transferred into sterile culture flasks and 18 ml complete medium containing 10% FBS was then added. Samples were then incubated at 37°C in a 5% CO_2_ incubator for 2 h of static culture. IFN-γ (1,000 U/ml) was added and continuously cultured for 24 h, 37°C, 5% CO_2_. IL-2 cytokines (300 U/ml) were then added with 50 ng/ml CD3 monoclonal antibody. Cells passaged every 3 days and a further 300 U/ml IL-2 was added with each passage. Samples were cultured at 37°C in a 5% CO_2_ incubator for 14 days to collect CIK cells. These cells were subsequently used for determining proliferation, cytotoxic activity and cytokine secretion.

### Proliferation assay of CAPRI cells and CIK cells

An inverted microscope was used to dynamically observe the proliferation of three PBMC suspensions (a, b and c), which were induced using the methods described above. On days 1, 3, 5 and 14 of culture, cells were stained using Trypan blue (Yocon Bio-Technology Co., Ltd., Beijing, China) and counted in order to determine proliferation and morphology. Experiments were performed in triplicate.

### Cytotoxic activity detection of effector cells using a lactate dehydrogenase (LDH) release assay

#### Cell culture groups

**Figure 1:**
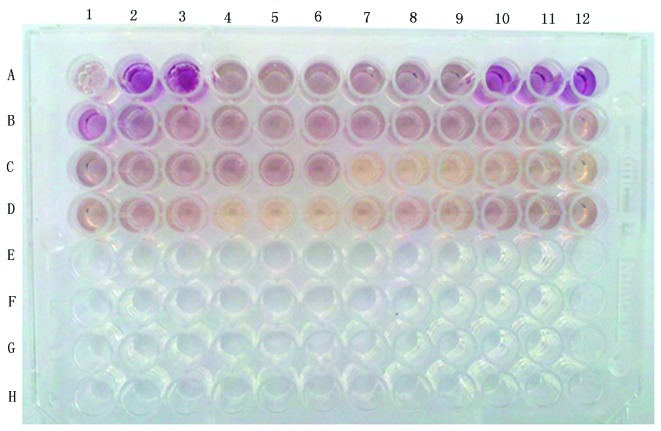
Schematic diagram of effector and target cell-plating (96-well plates) for the lacate dehydrogenase release assay.

CIK cells cultured for 14 days (a, b and c) and the thawed CAPRI cells (a, b and c) were the effector cells. K562 leukemia cells and MCF-7 breast cancer cells were used as the target cells. All experiments were performed in triplicate and plating of the treatment groups is shown in Fig. 1. The following procedure was performed separately for each of the two target cell lines.

Three different effector-target ratios of 40:1, 20:1, 10:1 were used; effector cells (100 *µ*l) concentrations of 4×10^6^, 2×10^6^ and 1×10^6^/ml were added to each well with 100 *µ*l target cells at a concentration of 1×10^5^/ml. CIK cells, B1-B9; and CAPRI cells, C7-C12 and D1-D3.

Effector cells (100 *µ*l/well) were plated at concentrations of 4×10^6^, 2×10^6^ and 1×10^6^/ml for each group (a, b and c) with 100 *µ*l culture medium in order to detect spontaneous cytotoxic activity. CIK cells, B10–B12 and C1–C6; and CAPRI cells, D3–D12.

In order to determine maximum cytotoxic activity, target cells (100 *µ*l/well) were plated at a concentration of 1×10^5^/ml into three wells (A1–A3) with 100 *µ*l culture medium. These cells were incubated at 37°C in a 5% CO_2_ culture incubator, at 45 min prior to the end of incubation, 20 *µ*l/well cell lysate was added. In addition, to determine the spontaneous cytotoxicity of target cells, 1×10^5^/ml target cells (100 *µ*l/well) were plated into three wells (A4–A6) with 100 *µ*l culture medium.

Blank control wells (A7–A9) consisted of 200 *µ*l/well culture medium; in addition, the corrected volume control wells consisted of 200 *µ*l/well culture medium (A10–A12), which were then incubated 20 *µ*l/well cell lysate, which was added at 45 min prior to the end of culture at 37°C in a 5% CO_2_ incubator.

#### Culture and supernatant collection

Following centrifugation at 250 × g for 4 min, cells were cultured at 37°C in a 5% CO_2_ incubator for 4–6 h. At 45 min prior to the end of culture, the culture plates were removed and 20 *µ*l cell lysate was added to wells in the target cells maximum cytotoxicity group and the corrected volume control group. Cells in these two groups were then centrifuged at 250 × g for 4 min and further cultured for 45 min.

#### LDH measurement

Each group was centrifuged at 250 × g for 4 min and 50 *µ*l supernatant per well was transferred into a separate 96-well plate with 50 *µ*l/well substrate mix and incubated for 30 min at room temperature in the dark. Stop solution (50 *µ*l/well; Yanhui Bio-Technology Co., Ltd., Shanghai, China) was then added and pigmented particles were broken up using an oscillator (G560E; Scientific Industries, New York, NY, USA). Absorbance was measured at 490 nm using an ELISA analyzer.

#### Results and calculation

Mean absorbance values were calculated for each group. In order to obtain the corrected values for A (test), T (target cells spontaneous release) and E (effector cells spontaneous release), the mean absorbance value for the blank control group was subtracted from the mean absorbance values for the test, target cells spontaneous release and effector cells spontaneous release groups. The mean absorbance values in target cell maximum release group minus the mean absorbance values in corrected volume group, and the corrected Tmax was obtained. Cytotoxic activity (%)=(A−E−T)/(Tmax−T)×100%.

#### ELISPOT detection of IFN-γ and IL-2 secretion

CAPRI cells (a1, b1 and c1) and CIK cells (a2, b2 and c2) were transferred and the stimulating substance was added, under aseptic conditions.

ELISPOT plates were precoated with 200 *µ*l/well RPMI 1640 medium and incubated at room temperature for 10 min. CAPRI or CIK cells at concentrations of 1×10^6^/ml and 5×10^5^/ml were then plated (100 *µ*l/well). The positive and negative control groups for each cell type were plated as follows: Negative control, 1×10^6^ and 5×10^5^ cells/ml with 10 *µ*l/well RPMI 1640 medium with 10% FBS; positive control, 1×10^6^ and 5×10^5^ cells/ml with phytohemagglutinin cell stimulator working fluid. The well plate was then covered and incubated at 37°C in a 5% CO_2_ incubator for 20 h. Following incubation, the cells and medium were removed and added to ice-cold deionized water (200 *µ*l/well). Hypotonic cell lysis was performed with Hypotonic Lysis Buffer (Amresco LLC, Solon, OH, USA) at 4°C for 10 min. Cells were then washed five times for 60 sec each with 1X washing buffer (200 *µ*l/well; Shenzhen Dakewei Biotechnology Co., Ltd.) and then dried on absorbent paper.

A biotin-labeled diluted antibody solution (100 *µ*l; in ELISPOT kit) was added to each experimental well and incubated at 37°C for 1 h. Cells were then washed, as above, and dried on absorbent paper. Horseradish peroxidase-avidin diluted antibody working solution (100 *µ*l) was added to each experimental well and incubated at 37°C for 1 h. Cells were then washed, as above, and dried on absorbent paper.

Each experimental well was stained with 3-amino-9-ethylcarbazole working solution (100 *µ*l/well; Shenzhen Dakewei Biotechnology Co., Ltd.) and incubated at room temperature in the dark for 25 min. In order to terminate staining, liquid was poured out in each well, the base plate was opened and deionized water (Xinyu, Shanghai, China) was used to wash the wells five times. The plates were then placed at room temperature in the dark, with the base closed, until they dried.

Spot count was then performed in the ELISPOT plates and various parameters of spots were recorded in order to determine IFN-γ and IL-2 secretion levels. Brown spots indicated that the cells had produced cytokines.

#### Statistical analysis

SPSS 17.0 software (SPSS, Inc., Chicago, IL, USA) was used for data analysis. Values are expressed as the mean ± standard deviation. Independent samples t-tests were used for comparisons between two groups. P<0.05 was considered to indicate a statistically significant difference between values.

## Results

### Morphology and cell proliferation activity of CAPRI cells and CIK cells

Cultured CAPRI cells began to proliferate within 2 days and entered the proliferation stage within 3 days. Following 14 days, cultured cells reached a density of (6.32±1.23)×10^7^. Cultured CIK cells began to proliferate within 3 days and entered the proliferation stage within 5 days. Following 14 days, cultured cells reached a density of (60.21±6.08)×10^7^. Following 3 days of culture, the proliferation rate of CIK cells was significantly faster compared with CAPRI cells; of note, at day 5, the proliferation rate of CIK cells was ~3 times that of CAPRI cells (P<0.001); at day 14, CIK cells had proliferated ~60 times compared with day 1, which was ~10 times that of CAPRI cells at day 14 (P<0.001) (Table I).

**Figure 2:**
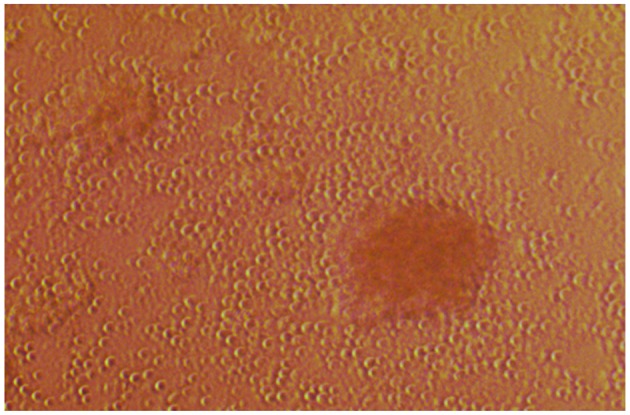
Representative image of cytokine-induced killer cell morphology at day 14 of culture (magnification, ×100).

**Figure 3:**
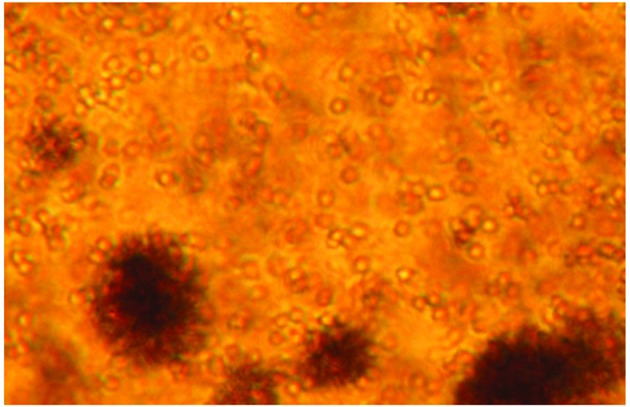
Representative image of cascade primed immune cell morphology at day 5 of culture (magnification, ×100).

Cells were harvested and examined under an inverted microscope, which revealed that cells grew as colonies in suspension (Figs. 2 and 3). Cell volume was markedly increased over time and the survival rate of cultured cells was >95%. Of note, CAPRI cell patent reagents instructions described that the cultured cells should be harvested for clinical treatment on day 5 (6); however, since the present study was a comparative to CIK cells, the experimental culture was performed for 14 days.

### Cytotoxic activity of CAPRI and CIK cells against K562 leukemia and MCF-7 breast cancer cells

The results of the cytotoxic activity of CAPRI and CIK cells against K562 cells are shown in Table II. These results demonstrated that the two cell types exhibited cytotoxicity on K562 cells. The cytotoxic activity of CAPRI cells were 55.1±3.25 and 35.0±2.65% at effector-target ratios of 40:1 and 20:1, respectively, which were significantly reduced compared with the corresponding values of the CIK cells, 60.0±3.03 and 39.7±3.42% (P=0.004 and 0.005, respectively). No significant difference was observed in cytotoxic activity at an effector-target ratio of 10:1 between the two groups (P=0.056).

The results of the cytotoxic activity of CAPRI and CIK cells against MCF-7 cells are shown in Table III. These results revealed that the two cell types exerted cytotoxic effects on MCF-7 cells. Cytotoxic activity of CAPRI cells were 71.5±3.06, 56.0±3.76 and 40.2±2.90% at effector-target ratios of 40:1, 20:1 and 10:1, respectively, which were significantly increased compared with the corresponding values of CIK cells, 65.4±3.86, 49.5±3.91 and 36.1±3.73% (P=0.002, 0.003 and 0.02, respectively).

### IFN-γ and IL-2 secretion levels of CAPRI and CIK cells

**Figure 4:**
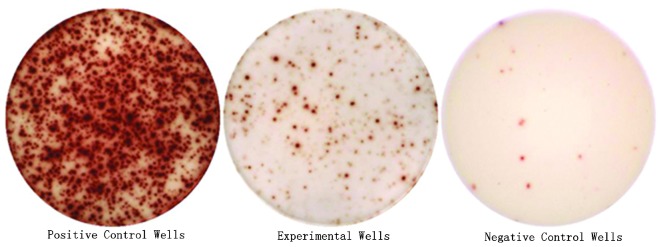
Representative images of cytokine enzyme-linked immunospot spot-forming cells (1×10^6^ cells/ml) for experimental, positive-control and negative-control groups (magnification, ×32). Interferon γ was detected here.

As shown in Fig. 4, each spot-forming cell (SFC) represented a measured cytokine secretion of CAPRI or CIK cells. ELISPOT analysis results are quantified in Tables IV and V. CAPRI and CIK cells each produced high levels of IFN-γ and IL-2 secretion. ELISPOT detection was performed at two different cell concentrations (1×10^6^ and 5×10^5^ cells/ml). In CAPRI cells, the number of IFN-γ SFCs detected were 126.2±10.31 and 48.8±10.99, respectively, which were significantly lower compared with the corresponding values in CIK cells, 409.3±7.76 and 159.3±15.45 (P<0.001) (Table IV). The number of IL-2 SFCs in CAPRI cells were 325.1±16.24 and 113.8±11.29 at concentrations of 1×10^6^ and 5×10^5^ cells/ml, respectively, which were increase compared with the corresponding values in CIK cells, 212.0±16.58 and 70.7±10.57 (P<0.001) (Table V).

## Discussion

Developments in the fields of molecular tumor biology, cell biology and immunology of cancer have resulted in a more comprehensive and in-depth understanding of tumors (9). Adoptive immunotherapy of cancer cells is a novel model of comprehensive treatment, which has an increasingly important role in cancer therapy (10). However, issues for the clinical use of this model require addressing, including differing effects of CAPRI and CIK cell therapies in different cell types, the balance between the use of adoptive immunotherapy and conventional treatments of surgery, chemotherapy and radiotherapy to achieve the highest combination efficacy as well as the method of activating the suppression status of the body’s immune system (11). CAPRI and CIK cell therapies are prominent adoptive cell immunotherapies, which are currently widely used in clinical practice (12).

CAPRI cells are chain autologous activated immune cells, the effector cells of which include natural killer (NK) cells, NK-like T cell lymphocytes (NKT cells), dendritic cells, CD4^+^ T helper (Th) cells and CD8^+^ cytotoxic T lymphocytes (CTLs) (6). Th cells and CTLs accounted for 80% of the major cytotoxic activity of CAPRI cells and the remaining effector cells accounted for 20% (6). CIK cells simultaneously express two types of membrane protein molecules, CD3^+^ and CD56^+^, and are also known as NKT cells; these cells were reported to have the oncolytic advantage of T lymphocytes as well as the non-major histocompatibility complex (MHC)-restricted antitumor activity of NK cells (13). The potent antitumor activities of CIK cells primarily proceed via the following four mechanisms: Direct cytotoxic effects on tumor cells (14); induced proliferation of T cells to CTL (15); induced apoptosis of tumor cells (16); and production of cytokines with oncolytic effects (17).

The antitumor mechanisms of CIK cells have been established; however, CAPRI cells are a novel method of treatment, for which the antitumor mechanisms are relatively undetermined (18). It was speculated that CAPRI cells were T lymphocytes, with comparable tumor cell-killing mechanisms to CIK cells. In the present study, a classic experiment was performed in order to compare the cytotoxic activity of CAPRI and CIK cells against K562 leukemia cells and MCF-7 breast cancer solid tumor cells. Previous studies have demonstrated that IFN-γ and IL-2 may be used alone in clinical treatment and as an inducer of cell therapy (19,20). Therefore, in the present study, the applications of these two cytokines were selected for ELISPOT technique detection in order to determine the indirect antitumor effect of CIK and CAPRI cells.

CAPRI cells were reported to require synergy of human leukocyte antigen (HLA)-I and HLA-II expression for successful tumor cell lyses (21). This demonstrated the complete interdependence of Th cells and CTLs. Generation of CTLs was demonstrated to be dependent on the interactions between α-β T cell receptors, peptide-MHCs and antigen-presenting cell surface molecules of HLA-II tumor immunogenic peptides (22). However, the complete dissolve of tumor cells may be dependent on the interaction of HLA-I- and HLA-II-type antigens (22). CAPRI cells were reported to enhance HLA-I and HLA-II expression on the surface of solid tumors; however, K562 cells were not induced to express leukocyte antigen and K562 cell lysis was primarily mediated by activated NKT cells in PBMC culture (23,24). The results of the present study demonstrated that CAPRI cells exerted potent and specific cytotoxic effects on breast cancer cells of solid tumors, whereas CIK cells had strong non-specific cytotoxic activity. These results may have practical significance for the clinical use of these two cell types for adoptive immunotherapy.

In the present study, ELISPOT analysis revealed that IFN-γ secretion levels of CIK cells were higher than those of CAPRI cells, whereas IL-2 secretion levels of CAPRI cells were higher than those of CIK cells. However, the secretion levels for IFN-γ and IL-2 were relatively high in the two cell types. CAPRI and CIK cells have different types of effector cells; therefore, their antitumor mechanisms may also differ. IL-2 is primarily produced by CD4^+^ and CD8^+^ T cells, which make up 80% of the CAPRI effector cells, while IFN-γ is predominantly produced by CD8^+^ T cells and NK cells, of which NK cells are the major effector cells of CIK cells (25). The cytotoxic mechanisms of the two cell types involve the tumor suppressing and cytotoxic effects of inhibitory cytokines, which are secreted by effector cells of CAPRI and CIK cells (26). The present study demonstrated that CAPRI and CIK exerted immunomodulatory effects via the secretion of IFN-γ and IL-2, which was an important aspect of the cytotoxic activity evaluation. The detection techniques used in the present study may be applied to clinical work, as patients may receive cytokines detection tests prior to and following adoptive cell immunotherapy in order to evaluate therapeutic effectiveness.

The key principle of tumor cell adoptive immunotherapy is to have sufficient quantities of immune cells with strong cytotoxic activity (27). These technical cycles are short (5 days) and have a high specificity; i addition, cells can be cryopreserved following harvesting, which provides convenience for clinical use.

The results of the current study demonstrated that the cell density of CAPRI and CIK cells was (6.32±1.23)×10^7^ and (60.21±6.08)×10^7^, respectively, following treatment for 14 days. The cell proliferative activity of CAPRI cells was significantly lower than that of CIK cells (P<0.001). Compared with the 1st day, the proliferative rate of CAPRI was 3 times higher at the 5th and 6 times higher at the 14th days, while CIK was 10 times higher at the 5th and 60 times higher at the 14th days.

In conclusion, the experimental results of the present study demonstrated that CAPRI cells have a weaker cytotoxic effect on NK target cells of K562 leukemia cells compared with their potent cytotoxic effect on the NK-insensitive solid tumors MCF-7 cells. These experimental methods may be used as a model to further explore the cytotoxic activity of tumor cells in other entities and to provide guidance for future clinical studies and treatment options. The cytotoxic activity of CIK cells is not MHC restricted, however CAPRI cells filter MHC of cells, which remedies the deficiency of CIK cells treatment. The ELISPOT technique may be used to detect the secretion of cytokines for these two cell types prior to and following treatment for clinical efficacy assessment. Since the culture time and antitumor mechanisms between CAPRI and CIK cells differed, this may offer the possibility for combination of the two cell types for antitumor therapy; however, further studies and clinical trials are required in order to confirm the effectiveness of this combined therapy.

## Figures and Tables

**Table I:** Proliferation comparison of CAPRI cells and CIK cells.

Day of culture	CAPRI cells (×10^7^)	CIK cells (×10^7^)	t-value	P-value
1	1.21±0.21	1.16±0.20	0.447	0.661
3	2.33±0.56	3.19±1.14	2.033	0.059
5	3.61±1.16^a^	10.39±4.24	4.629	<0.001
14	6.32±1.23^a^	60.21±6.08	26.057	<0.001

Values are presented as the mean ± standard deviation (n=9).

a P<0.05 vs. CIK cells. CAPRI cells, cascade primed immune cells; CIK, cytokine-induced killer cells.

**Table II:** Cytotoxic activity of CAPRI cells and CIK cells against K562 leukemia cells.

Effector-target ratio	CAPRI cells (%)	CIK cells (%)	t-value	P-value
40:1	55.1±3.25^a^	60.0±3.03	3.310	0.004
20:1	35.0±2.65^a^	39.7±3.42	3.265	0.005
10:1	28.5±2.36	30.6±1.72	2.062	0.056

Values are presented as the mean ± standard deviation (n=9).

a P<0.05 vs. CIK cells. CAPRI cells, cascade primed immune cells; CIK, cyto-kine-induced killer cells.

**Table III:** Cytotoxic activity of CAPRI cells and CIK cells against MCF-7 breast cancer cells.

Effector-target ratio	CAPRI cells (%)	CIK cells (%)	t-value	P-value
40:1	71.5±3.06^a^	65.4±3.86	3.729	0.002
20:1	56.0±3.76^a^	49.5±3.91	3.563	0.003
10:1	40.2±2.90^a^	36.1±3.73	2.582	0.020

Values are presented as the mean ± standard deviation (n=9).

a P<0.05 vs. CIK cells. CAPRI cells, cascade primed immune cells; CIK, cytokine-induced killer cells.

**Table IV:** Number of IFN-γ spot-forming cells at different cell densities of CAPRI cells and CIK cells.

Cell density	CAPRI cells	CIK cells	t-value	P-value
1×10^6^/ml	126.2±10.31^a^	409.3±7.76	65.833	<0.001
5×10^5^/ml	48.8±10.99^a^	159.3±15.45	17.494	<0.001

Values are presented as the mean ± standard deviation (n=9).

a P<0.05 vs. CIK cells. CAPRI cells, cascade primed immune cells; CIK, cytokine-induced killer cells; IFN-γ, interferon-γ.

**Table V:** Number of IL-2 spot-forming cells at different cell densities of CAPRI cells and CIK cells.

Cell density	CAPRI cells	CIK cells	t-value	P-value
1×10^6^/ml	325.1±16.24^a^	212.0±16.58	14.621	<0.001
5×10^5^/ml	113.8±11.29^a^	70.7±10.57	8.362	<0.001

Values are presented as the mean ± standard deviation (n=9).

a P<0.05 vs. CIK cells. CAPRI cells, cascade primed immune cells; CIK, cytokine-induced killer cells; IL-2, interleukin-2.
